# Chaperone-mediated MHC-I peptide exchange in antigen presentation

**DOI:** 10.1107/S2052252524002768

**Published:** 2024-04-24

**Authors:** Jiansheng Jiang, Kannan Natarajan, David H. Margulies

**Affiliations:** a Molecular Biology Section, Laboratory of Immune System Biology, National Institute of Allergy and Infectious Diseases, Bethesda, MD 20892, USA; University of Michigan, USA

**Keywords:** structural immunology, antigen presentation, major histocompatibility complex, MHC, peptide exchange, PLC, MHC-I/tapasin, MHC-I/TAPBPR, chaperones

## Abstract

This work focuses on molecules that are encoded by the major histocompatibility complex (MHC) and that bind self-, foreign- or tumor-derived peptides and display these at the cell surface for recognition by receptors on T lymphocytes (T cell receptors, TCR) and natural killer (NK) cells. The past few decades have accumulated a vast knowledge base of the structures of MHC molecules and the complexes of MHC/TCR with specificity for many different peptides. In recent years, the structures of MHC-I molecules complexed with chaperones that assist in peptide loading have been revealed by X-ray crystallography and cryogenic electron microscopy. These structures have been further studied using mutagenesis, molecular dynamics and NMR approaches. This review summarizes the current structures and dynamic principles that govern peptide exchange as these relate to the process of antigen presentation.

## Abstract 2

This topic review summarizes structures of chaperones complexed with MHC-I, the structural principles that govern peptide exchange and the mechanism in antigen presentation.

## 1. Introduction

Structural immunology has progressively developed for over 50 years (Wilson & Stanfield, 2021 ▸) since the biochemical and structural characteristics of antibodies were first addressed (Tiselius & Kabat, 1939 ▸; Edelman, 1973 ▸; Porter, 1967 ▸). The field now extends to a host of molecules and complexes that function in the immune system and beyond, such as antibodies, antigen receptors, cytokines and their receptors, T cell receptors (TCR), and natural killer (NK) cell receptors. Molecules that play a role in antigen presentation, such as MHC Class I or II, CD1, MR1, and other surface recognition molecules such as Toll-like receptors have been studied. Various molecules derived from pathogens such as viral immunoevasins have also been studied in structural detail. Somatic cells of the immune system participate in immunological processes that regulate immunity to various pathogens and the resistance or susceptibility to cancer and autoimmunity. Immunological experiments explore many immune responses, pathways, regulation, recognition and specificity, providing valuable data for therapeutic manipulation. Structural determination (primarily by X-ray crystallography or cryoEM) of ligand–receptor complexes and various multiprotein complexes such as the peptide-loading complex (PLC) continue to elucidate details of interactions that assist immunologists and medical scientists in understanding functions and mechanisms better. A significant goal of structural immunology is to exploit such structural information to generate drugs, treatments, immunogens and vaccines.

This review focuses on the highly polymorphic MHC-I molecules that play a critical role in immunity. MHC-I molecules load self-, foreign- (pathogen) or tumor-derived peptides and presents them at the cell surface for recognition by TCR and NK cell receptors (Blum *et al.*, 2013 ▸; Margulies, Natarajan *et al.*, 2023 ▸; Rock *et al.*, 2016 ▸). Precisely how MHC-I molecules selectively load high-affinity peptides is a question that has puzzled immunologists for several decades. A vast knowledge base is now available of the structures of MHC molecules bound to numerous peptides and TCRs with specificity for many different peptide/MHC (pMHC) complexes. Our understanding of how peptides are loaded and exchanged on MHC molecules has matured in recent years with the structural definition of MHC-dedicated chaperones and their interactions with MHC. Beginning with the determination of the structure of the chaperone tapasin bound to another stabilizing component ERp57 (also known as PDIA3) (Dong *et al.*, 2009 ▸), several additional structures determined by X-ray crystallography (Jiang *et al.*, 2017 ▸; Thomas & Tampé, 2017 ▸; Müller *et al.*, 2022 ▸; Jiang *et al.*, 2022 ▸) or cryoEM (Blees *et al.*, 2017 ▸; Domnick *et al.*, 2022 ▸) have augmented a structure-based mechanistic view of chaperone function. Several recent review papers on this topic are primarily based on a biological perspective (Margulies *et al.*, 2022 ▸; Margulies, Jiang *et al.*, 2023 ▸; Turner *et al.*, 2023 ▸; van Hateren & Elliott, 2023 ▸; Lan *et al.*, 2023 ▸; Satti *et al.*, 2023 ▸). This review will emphasize a structural point of view (Table 1 ▸) and the principles governing peptide exchange.

## 2. MHC molecules and antigen presentation pathways

MHC molecules represent proteins found on the surface of nucleated vertebrate cells. Initially identified for their role in graft rejection, these molecules are recognized as the most polymorphically known. These molecules play a crucial role in the antigen presentation pathway by binding antigenic peptides and displaying them at the cell surface to T cells and NK cells for initiating and regulating immune responses.

### 2.1. MHC molecules, peptides and structures

MHC molecules are designated as Class I, Class II or non-classical MHC-I (or MHC-I-like, such as CD1, MR1) based on their gene/protein sequences (Margulies, Natarajan *et al.*, 2023 ▸). In humans, the MHC genes and their encoded proteins are referred to as human leukocyte antigens (HLA), and the genetic loci are designated HLA-A, -B, -C, -E, -F and -G for Class I and HLA-D for Class II. Orthologous MHC genes in mice are called H2 (or H-2), and the proteins are designated H2-K, -D or -L. Different alleles are designated as HLA-A*01, HLA-A*02 *etc.* with more precise discriminators by sequence, such as HLA-A*01:01 (Marsh, 2019 ▸; Margulies, Natarajan *et al.*, 2023 ▸). In mice, due to the availability of syngeneic lines, allelic designation is by haplotype (*e.g.* *b*, *d*, *k*, *s* *etc.*), written as an italicized superscript. Some 26 000 human MHC-I (HLA-I) and 11 000 MHC-II (HLA-II) genes have been identified and are tabulated in the Immuno Polymorphism Database (IPD) (Barker *et al.*, 2023 ▸) and International Immunogenetics Information System (IMGT) (Lefranc *et al.*, 1999 ▸).

**Figure 1:**
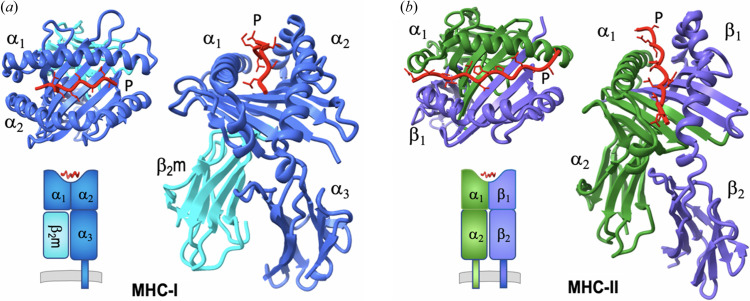
MHC molecules and structures. (*a*) MHC-I structure and domains (PDB entry 3mre; Reiser *et al.*, 2014 ▸). (*b*) MHC-II structure and domains (PDB entry 3c5j; Dai *et al.*, 2008 ▸). Illustrations from PDB coordinates and EMD maps were prepared with *PyMOL* (version 2.5.4, Schrödinger) and *ChimeraX* (Pettersen *et al.*, 2021 ▸).

MHC-I molecules consist of a membrane-anchored α or heavy chain (∼40 kDa) and a smaller light chain (∼12 kDa) designated β_2_-microglobulin [β_2_m; Fig. 1 ▸(*a*)]. MHC-II molecules consist of two chains approximately equal in size: an α chain and a β chain (about 30–33 kDa) [Fig. 1 ▸(*b*)], both anchored to the membrane. A typical ‘MHC fold’ consists of a 90 amino acid long polypeptide chain that folds into an α-helix and 3–4 antiparallel β-strands supporting the helix (Saper *et al.*, 1991 ▸). Two MHC folds symmetrically construct a 7–8 stranded β-sheet ‘bed’ flanked by two antiparallel α-helices, designated α1 and α2 for MHC-I, or α1 and β1 for MHC-II, forming a platform domain that contains the peptide-binding groove. The MHC-I platform is connected to a membrane-proximal Ig-like domain designed α3 followed by the transmembrane segment and a short cytoplasmic tail. As noted above, MHC-I molecules are non-covalently associated with the β_2_m chain [Fig. 1 ▸(*a*)], a soluble single-domain Ig-fold protein that provides structural stability. By contrast, the platform domain of MHC-II is formed by α1 and β1 domains on the top, and α2 and β2 Ig-like domains both connect to the membrane [Fig. 1 ▸(*b*)]. A common feature of MHC molecules is that peptide is bound in the groove of the platform domain. The length of peptides is usually 8–10 for MHC-I and 12–20 for MHC-II. The binding groove of MHC-like molecules (*e.g.* CD1 and MR1) is modified to bind non-peptidic ligands such as glycolipids, metabolites or drugs. The bound peptide is crucial for refolding and structural stability of the MHC molecule. Because of the ability of any particular MHC molecule to bind a large number of peptides with a characteristic binding motif, a large number (about 1.5 million) of possible epitopic peptides have been identified and collected in the IEDB (Vita *et al.*, 2019 ▸). About 3500 structures of MHC (∼2000 MHC-I, ∼1000 MHC-II) and their complexes (∼500 TCR/MHC) are available (Jiang *et al.*, 2019 ▸) in the Protein Data Bank (PDB) (Berman *et al.*, 2002 ▸) and IMGT3D (Kaas *et al.*, 2004 ▸).

### 2.2. Classical antigen presentation pathways

**Figure 2:**
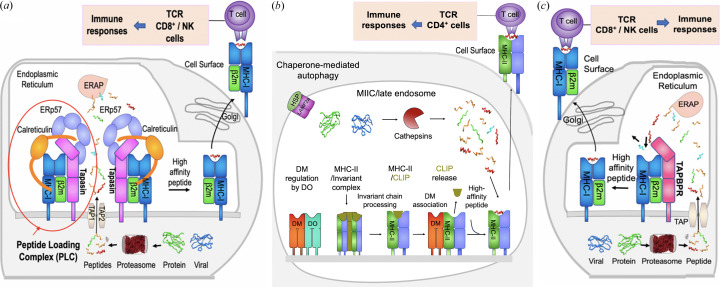
Chaperone/MHC and antigen presentation pathways. (*a*) MHC-I/tapasin classical antigen presentation pathway. (*b*) MHC-II/DM classical antigen presentation pathway (Blum *et al.*, 2013 ▸). (*c*) MHC-I/TAPBPR auxiliary antigen presentation pathway.

Cell surface MHC antigen presentation is crucial in adaptive immunity (Rock *et al.*, 2016 ▸; Blum *et al.*, 2013 ▸; Pishesha *et al.*, 2022 ▸). The process of generating antigenic peptides, proper folding of MHC molecules, loading of peptides onto MHC molecules, transporting to the cell surface and presentation of MHC-bound peptide antigens to T cells for recognition of foreign and dysregulated antigens is known as ‘antigen processing and presentation.’ Fig. 2 ▸(*a*) illustrates the classical antigen presentation pathway for MHC-I. The proteasome, a multiprotein organelle, digests self, viral or bacterial proteins, which are then delivered to the endoplasmic reticulum (ER) via ATP-dependent transport proteins TAP1/TAP2 (TAP). Peptide loading onto MHC-I occurs within the ER in the peptide-loading complex (PLC), which consists of TAP, tapasin [also known as TAP binding protein (TAPBP)], MHC-I, ERp57 and calreticulin. The endoplasmic reticulum amino-peptidase (ERAP1 and 2 in humans or ERAAP in mice) may further trim the peptides to an optimal length for MHC-I loading. Tapasin plays a chaperone role for MHC-I by stabilizing the PLC and facilitating selective loading/exchanging of high-affinity peptides (Chen & Bouvier, 2007 ▸). When MHC-I is loaded with a high-affinity peptide, it dissociates from tapasin (Rizvi & Raghavan, 2006 ▸) and the PLC and then is transported to the cell surface, where CD8^+^ T cells or NK cells can recognize the peptide-MHC-I complex (pMHC) and trigger an immune response against tumors or infected cells. Although this general pathway of MHC-I loading has been known for more than three decades, insights into the mechanism of how the high-affinity peptide is selected and exchanged with the help of chaperone were recently clarified when the tapasin/MHC-I complex structures were solved (see later sections).

In the antigen presentation pathway of MHC-II [Fig. 2 ▸(*b*)], the chaperone is DM [HLA-DM for humans, H2-DM for mice (Mellins & Stern, 2014 ▸)] and peptide loading occurs in endosomes. MHC-II is first stabilized by association with invariant chain (Ii) (Cresswell & Roche, 2014 ▸; Landsverk *et al.*, 2011 ▸), which is later processed to a class II-associated invariant chain peptide (CLIP), a short and low-affinity peptide that stabilizes the peptide-binding groove. The chaperone, DM, functions for MHC-II much like tapasin does for MHC-I by stabilizing MHC-II on release of CLIP until MHC-II loads with higher-affinity peptide (Pos *et al.*, 2012 ▸). DO (HLA-DO for humans, H2-DO for mice) regulates DM for peptide loading (Guce *et al.*, 2012 ▸). MHC-II loaded with high-affinity peptide dissociates from DM and traffics to the cell surface [Fig. 2 ▸(*b*)] to be recognized by TCRs on CD4^+^ T cells (Blum *et al.*, 2013 ▸; Jurewicz *et al.*, 2019 ▸).

### 2.3. Auxiliary antigen presentation pathway

A gene encoding a homolog of tapasin, known as TAP-binding protein-related TAPBPR, was identified (Teng *et al.*, 2002 ▸) and the protein was shown to be a molecule with a function similar to that of tapasin (Boyle *et al.*, 2013 ▸; Hermann *et al.*, 2015 ▸; Morozov *et al.*, 2016 ▸). The sequence of TAPBPR is only 22% identical to that of tapasin, but it shares structural domain organization. A small-angle X-ray scattering (SAXS) study of TAPBPR revealed envelope density similar to that of tapasin (Morozov *et al.*, 2016 ▸), and TAPBPR showed a higher binding affinity for MHC-I molecules emptied of peptides than those bound to peptides (Boyle *et al.*, 2013 ▸; Morozov *et al.*, 2016 ▸). Thus, TAPBPR, as an MHC-I chaperone, is an additional player in the antigen presentation pathway [Fig. 2 ▸(*c*)], although TAPBPR is not a component of the PLC. The structural studies of TAPBPR/MHC-I showed that TAPBPR interacts with MHC-I via the same broad interface as tapasin (Jiang *et al.*, 2017 ▸; Thomas & Tampé, 2017 ▸) (see below).

## 3. Strategies to obtain complexes of MHC-I with chaperones

Because of the well known instability of MHC-I molecules lacking bound peptides, several strategies have been employed to first obtain homogeneous peptide/MHC-I/β_2_m complexes, and then to generate a metastable state capable of binding a chaperone. Two approaches were considered: (1) to refold MHC-I with a photo-labile peptide that could be cleaved by UV-irradiation (Toebes *et al.*, 2006 ▸) before binding to the chaperone, or (2) to refold MHC-I with a truncated low-affinity peptide mimicking sub-optimally loaded MHC-I.

For example, HLA-A2 was refolded with a photo-sensitive peptide, photo-FluM1 (GILGFVFJ*L), which contained 3-amino-3-(2-nitro)­phenyl-propionic acid (designated J*) in place of threonine at position 8, and following mixture with TAPBPR, the putative complex was irradiated with UV at 366 nm. Native gel electrophoresis and size-exclusion chromatography confirmed the formation of a TAPBPR/HLA-A2 complex (Morozov *et al.*, 2016 ▸). This photo-cleavable peptide strategy was used to form complexes of TAPBPR/H2-D^b^ (Thomas & Tampé, 2017 ▸) and tapasin/ERp57/H2-D^b^ (Müller *et al.*, 2022 ▸) with the photo-P18-I10 (RGPGRAFJ*TI). Such a strategy was also used to examine the binding grooves of MHC-II and HLA-DR1 (Negroni & Stern, 2018 ▸).

**Figure 3:**
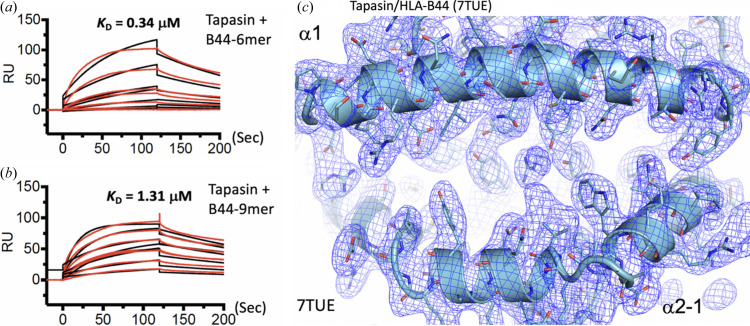
Strategies for obtaining the chaperone-mediated MHC-I complex. (*a*) Surface plasmon resonance (SPR) experiments: B44/6-mer binds better (*K*_D_ = 0.34 µ*M*) than (*b*) that of B44/9-mer (*K*_D_ = 1.31 µ*M*), which indicates that MHC-I with the short/truncated peptide binds tighter to tapasin. (*c*) No electron density is observed in the peptide groove of HLA-B44:05 in the complex with tapasin (PDB entry 7tue). The electron density of the 2(*F*_o_ − *mF*_c_) map is shown in blue, contoured at 1σ.

An alternative method to obtain refolded MHC-I molecules with a partially empty binding groove was developed. MHC-I molecules with a cysteine substitution of residue 73 of the α1-helix were engineered and these molecules were refolded with truncated peptides containing a cysteine at each of several positions. A similar strategy had proven helpful for MHC-II molecules to isolate HLA-DM/HLA-DR1 complexes (Pos *et al.*, 2012 ▸). A series of C-terminal truncations of the peptide (length from 10 to 5) were tested in refolding MHC-I (both H2-D^d^ and HLA-B44). A dipeptide was added during the refolding to stabilize a region of the binding groove that accommodates the C-terminal side chain of the bound peptide, known as the F-pocket (Saini *et al.*, 2013 ▸). Surface plasmon resonance studies indicated that tapasin binds to an HLA-B*44/6-mer (*K*_D_ = 0.34 µ*M*) better than to an HLA-B*44/9-mer (*K*_D_ = 1.31 µ*M*) [Figs. 3 ▸(*a*) and 3 ▸(*b*)], and TAPBPR binds to an H2-D^d^/5-mer (*K*_D_ = 0.009 µ*M*) better than to an H2-D^d^/10-mer (*K*_D_ = 0.19 µ*M*) (Jiang *et al.*, 2017 ▸). This peptide trap using truncated peptides aims to (1) stabilize the peptide receptive state of MHC-I and (2) increase the α2–1 helix dynamics to support interaction with the chaperone. The formation of the di­sulfide bond between the MHC and peptide cysteines depends primarily on the distance between the two S atoms achieved in refolding the MHC-I protein with the peptide. This type of di­sulfide bond may be sensitive to cleav­age by X-ray radiation (Weik *et al.*, 2000 ▸; Bhattacharyya *et al.*, 2020 ▸). Crystals of TAPBPR/H2-D^d^ and tapasin/HLA-B*44:05 complexes were obtained using this strategy. Structure determination of the complexes revealed no remaining electron density representing peptide in the binding grooves, as shown in the example of tapasin/HLA-B*44:05 [Fig. 3 ▸(*c*)] (Jiang *et al.*, 2022 ▸, 2017 ▸).

Earlier X-ray crystal structures of MHC-I molecules complexed with UV-irradiated peptides had already shown that some peptide residues remained in the peptide-binding groove (Celie *et al.*, 2009 ▸). Additional studies from the Springer laboratory suggested that various dipeptides added directly to refolding buffers could promote proper folding (Saini *et al.*, 2013 ▸; Anjanappa *et al.*, 2020 ▸). However, these approaches had not yet been tested for binding with chaperone. Both strategies described above were designed to allow refolding of the MHC-I molecule and binding to the chaperone but to reveal the effects of partial occupancy of the peptide groove in binding to the chaperone. Fragments of photo-lysed peptides may remain in the groove or be released during the formation of the TAPBPR or tapasin-containing complex, or during purification and crystallization. The di­sulfide-linked truncated peptides might assume variant conformations or be liberated with the crystals’ exposure to X-ray-irradiation. The efficiency of removing a low-affinity peptide or a peptide fragment from the groove depends on the function of the chaperone and the resulting conformational changes in the groove. Recently, others have engineered di­sulfide-stabilized MHC-I molecules linking the heavy chain and β_2_m and demonstrated their utility in peptide loading (Sun, Papadaki *et al.*, 2023 ▸; Sun, Young *et al.*, 2023 ▸). Tapasin can bind to such ‘empty’ MHC-I (HLA-B*37:01) in solution (Sun, Young *et al.*, 2023 ▸; Sun, Papadaki *et al.*, 2023 ▸). Solution NMR studies verify this type of ‘empty’ groove of MHC-I molecules. However, obtaining three-dimensional structures in such ultimately ‘empty’ MHC-I molecules remains challenging in the absence of chaperones.

## 4. Complexes of MHC-I with the chaperone

The first X-ray crystal structure of tapasin was determined as a complex of tapasin with another chaperone component of the PLC, ERp57, which revealed the di­sulfide-linked heterodimer (Dong *et al.*, 2009 ▸). Mutagenesis of tapasin and binding studies depicted the possible interaction sites with MHC-I. In recent years, two TAPBPR/MHC-I structures were determined (Jiang *et al.*, 2017 ▸; Thomas & Tampé, 2017 ▸). One structure of tapasin/MHC-I was solved (Jiang *et al.*, 2022 ▸) and the structure of a heterotrimer of tapasin/ERp57/MHC-I was determined (Müller *et al.*, 2022 ▸). The complete PLC structure was first revealed in a cryoEM map at a resolution of 5.8 Å (Blees *et al.*, 2017 ▸), and subsequently the map was improved to 3.7 Å (Domnick *et al.*, 2022 ▸) as summarized in Table 1 ▸.

### 4.1. The TAPBPR/MHC-I complex

**Figure 4:**
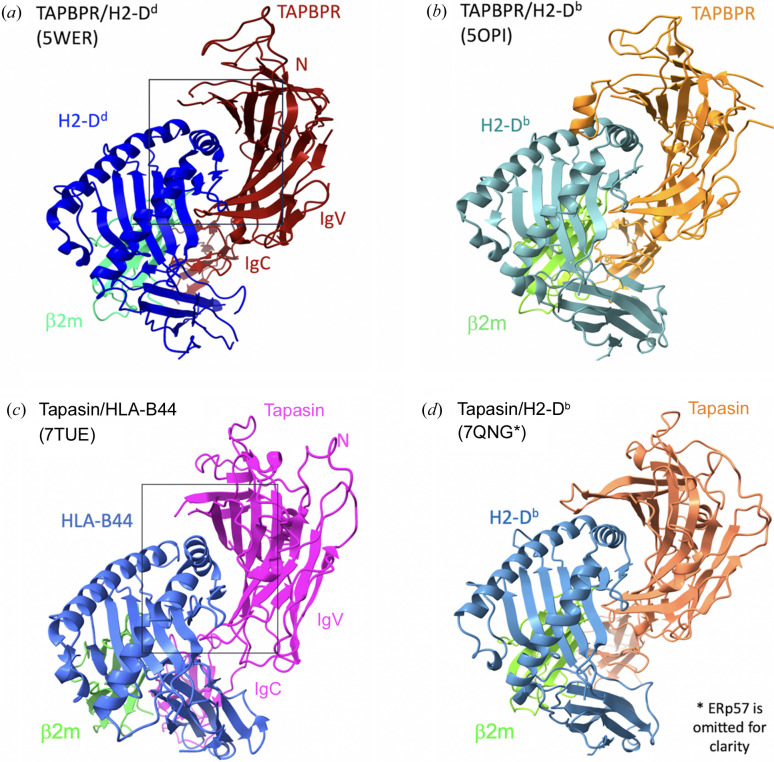
Overall structures of MHC-I with the chaperones. (*a*) Structure of TAPBPR/H2-D^d^ (PDB entry 5wer; Jiang *et al.*, 2017 ▸). (*b*) Structure of TAPBPR/H2-D^b^ (PDB entry 5opi; Thomas & Tampé, 2017 ▸). (*c*) Structure of tapasin/HLA-B44 (PDB entry 7tue; Jiang *et al.*, 2022 ▸). (*d*) Structure of tapasin/H2-D^b^/ERp57 (PDB entry 7qng; Müller *et al.*, 2022 ▸) where ERp57 has been omitted for clarity.

**Figure 5:**
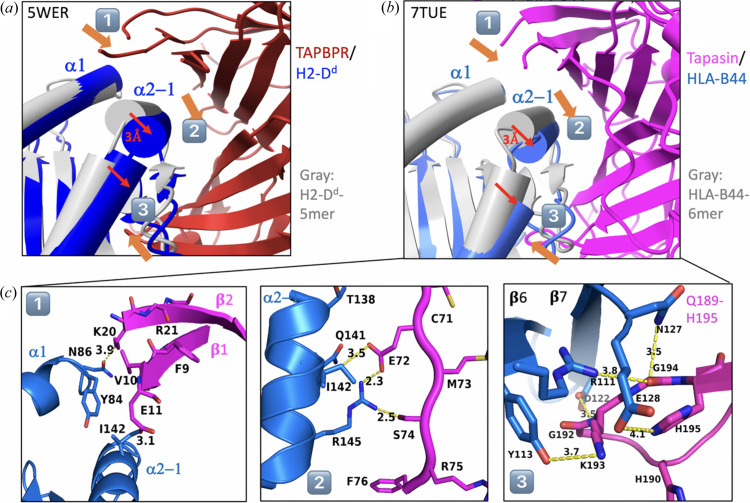
Peptide groove comformational changes and three major interaction sites. (*a*) TAPBPR/H2-D^d^ (PDB entry 5wer, blue and firebrick) is superimposed with H2-D^d^-5-mer (PDB entry 5wes, gray). Three major interaction sites are numbered. (*b*) Tapasin/HLA-B44 (PDB entry 7tue, marine blue and magenta) is superimposed with HLA-B44-6-mer (PDB entry 7tud, gray). Tapasin draws the α2–1 helix of HLA-B44 closer by about 3.0 Å and results in the groove being open. The three major interaction sites are numbered, and shown in detail in (*c*).

The structure of TAPBPR/MHC-I was solved independently in two laboratories in 2017 (Jiang *et al.*, 2017 ▸, Thomas & Tampé, 2017 ▸). The structures (PDB entries 5wer and 5opi, respectively) are broadly similar [Figs. 4 ▸(*a*) and 4(*b*)]. TAPBPR cradles the MHC-I, nestling with the dynamic α2–1 helix. Strikingly, the resulting maps lack electron density in the peptide groove, indicating that the peptide was lost and that the MHC-I presents a peptide receptive state when bound to TAPBPR. In comparison with unchaperoned MHC-I, the α2–1 helix was drawn away towards TAPBPR and the peptide groove was relaxed [Fig. 5 ▸(*a*)], with the Tyr84 side chain of the MHC-I flipping towards the outside of the groove and contacting Glu102 of TAPBPR. The α3 domain, β_2_m subunit and IgC domain of TAPBPR are reoriented in the complex. Both complex structures show these similarities even though the MHC-I allele differs (H2-D^d^ in PDB entry 5wer and H2-D^b^ in PDB entry 5opi). The structural characteristics of the TAPBPR/MHC-I complexes reflect the chaperone function of TAPBPR on MHC-I. It stabilizes the peptide-receptive state by changing the conformation of the peptide groove and allowing the release of low-affinity (sub-optimal) peptide. In PDB entry 5wer (Jiang *et al.*, 2017 ▸), there are four copies of the complex in the asymmetric unit; each complex may present different states of dynamic motion (Margulies *et al.*, 2020 ▸). The electron density of the loop (residues 25–34) linking the β1 and β2 strands was missing, which may be due to ‘intrinsic disorder’ or dynamic movement. Consequently, the loop model was not built. However, for PDB entry 5opi (Thomas & Tampé, 2017 ▸), this region was modeled as a short helix (this loop will be discussed later).

### 4.2. The tapasin/MHC-I complex

**Figure 6:**
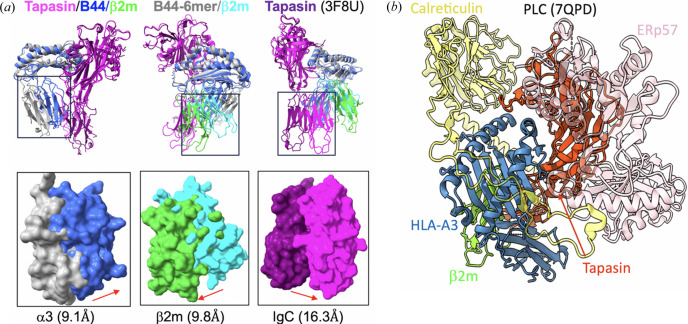
Tapasin/MHC-I domain movements and PLC. (*a*) Tapasin/B44:05, B44-6-mer and tapasin (PDB entry 3f8u) superimposed on the upper domains. Surface representation for the domains of α3, β_2_m and IgC are observed moving about 9.1, 9.8 and 16.3 Å, respectively. (*b*) Model of PLC, cryoEM map resolution at 3.7 A (PDB entry 7qpd; EMD-14119; Domnick *et al.*, 2022 ▸).

Obtaining a complex of tapasin/MHC-I was more challenging than TAPBPR/MHC-I because the binding affinity of tapasin to MHC-I is much lower than that of TAPBPR to MHC-I. After several attempts, we obtained crystals of tapasin complexed with the human MHC-I molecule HLA-B*44:05 using the same strategy of refolding HLA-B*44:05 containing a covalently linked short peptide (6-mer), and the structure was solved at 3.1 Å resolution (PDB entry 7tue; Jiang *et al.*, 2022 ▸). The overall structure [Fig. 4 ▸(*c*)] and the binding interface are similar to those of TAPBPR/MHC-I. Compared with unchaperoned HLA-B*44:05/6-mer (PDB entry 7tud; Jiang *et al.*, 2022 ▸), a large displacement (∼3 Å) of the α2–1 helix and the β8 strand, resulting in opening of the groove, is observed [Figs. 5 ▸(*a*) and 5 ▸(*b*)]. The peptide groove is widened and deepened. Arg145 of HLA-B*44:05 forms hydrogen bonds to both Glu72 and Ser74 of tapasin [Fig. 5 ▸(*c*)]. Remarkably, no electron density was observed in the peptide-binding groove, suggesting that peptide was lost [Fig. 3 ▸(*a*)]. Meanwhile, the loop Gln189–His195 of tapasin binds beneath the platform domain (strands β6 and β7), stabilizing the peptide-binding groove [Fig. 5 ▸(*c*)]. Also, the α3 domain, β_2_m and the IgC domain of tapasin show large domain movements (9–14 Å) [Fig. 6 ▸(*a*)]. The conformational changes and domain movements of HLA-B*44:05 suggest that tapasin interacts with MHC-I to create and stabilize a peptide-receptive state, poised to exchange an optimal peptide. Also, a structure of the heterotrimer of tapasin/ERp57/H2-D^b^ (PDB entry 7qng) was determined (Müller *et al.*, 2022 ▸) [Fig. 4 ▸(*d*)]. Compared with the unliganded H2-D^b^, it revealed the same conformational changes of the peptide-binding groove. The IgC domain of tapasin is also twisted compared with its position in tapasin/ERp57 (PDB entry 3f8u).

### 4.3. CryoEM structures of PLC

As noted above, the PLC is a multiple-component molecular complex in the ER, consisting of the transporter associated with antigen processing (TAP), TAP1/2 heterodimer, tapasin, MHC-I, ERp57 and calreticulin. MHC-I plays a central role in the PLC [Fig. 2 ▸(*a*)]. The membrane-embedded protein TAP pumps peptides from the cytoplasm into the ER. Tapasin, the chaperone of MHC-I, facilitates peptide loading and exchange and stabilizes the PLC. ERp57, a thiol oxido­reductase with a di­sulfide linkage to tapasin, and calreticulin, a lectin, contribute to the recruitment of MHC-I to the PLC. A PLC model was proposed previously (Dong *et al.*, 2009 ▸), and the PLC cryoEM map at 5.8 Å resolution (6eny, EMD-3906) was consistent with that model (Blees *et al.*, 2017 ▸), revealing a well organized architecture for peptide loading. Further improvement of the cryoEM map to 3.7 Å resolution (7qpd, EMD-14119) [Fig. 6 ▸(*b*)] revealed allosteric coupling between the MHC-I assembly and glycan processing (Domnick *et al.*, 2022 ▸). In both reported PLC cryoEM structures, the MHC-I is HLA-A*03.

## 5. From structures to mechanism

The recently determined structures of chaperone/MHC-I complexes provide a more complete model of the general mechanism of peptide exchange in antigen presentation.

### 5.1. Flexibility of dynamic loops and domain movements of the chaperone

**Figure 7:**
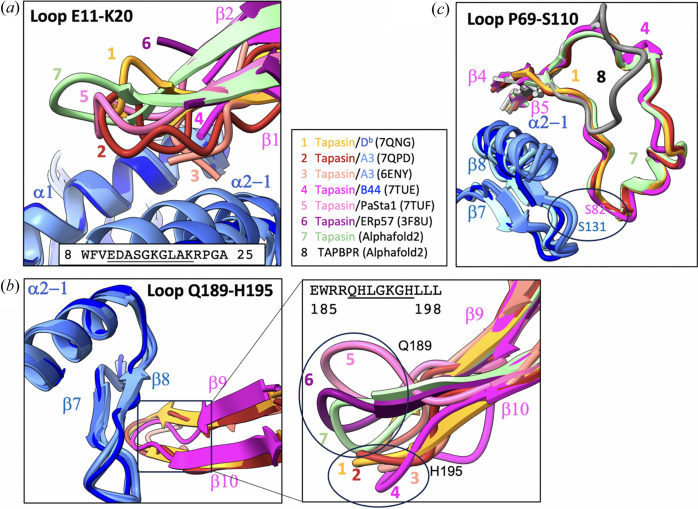
Flexibility of dynamic loops. (*a*) Tapasin loop E11–K20 modeled from various complexes is superimposed on the α1 helix. MHC-I is blue and the tapasin of each complex is color-coded according to the legend in the panel [PDB entries 3f8u (6), 7tue (4) and 6eny (3) are missing a number of residues on the loop]. The conformation of the loop varies indicating the mobility and flexibility of this loop. The α2–1 helices are drawn towards tapasin in various degrees indicating the openness of peptide groove in different structures. (*b*) Tapasin loop Q189–H195, the complexes are superimposed on α2–1. The loop interacts with β7 and β8 underneath the peptide-binding groove. Inset: tapasin loop from PDB entries 7tuf and 3f8u, the loop occupies a higher position. When tapasin is complexed with MHC-I (*e.g.* PDB entries 7qng, 7qpd, 7tue and 6eny), the loop is pushed down by 5–10 Å. (*c*) Tapasin loop P69–S110 (color) and TAPBPR loop C101–Q126 (gray). Tapasin has a much longer loop P69–S110 (40 aa) than that of TAPBPR (26 aa) which increased the interaction between the β8 loop and S82–K84 of tapasin.

We have observed several loops of the chaperones that lack electron density, *i.e.* the loops Ala25–Glu34 of TAPBPR/MHC-I (PDB entry 5wer) and the analogous Glu11–Lys20 in the complex of tapasin/MHC-I (PDB entry 7tue). We consider that the missing electron density in the loop Ala25–Glu34 of TAPBPR is due to dynamic movements or ‘intrinsic disorder’. By superimposing the α1 helix of MHC-I, we compared the loop Glu11–Lys20 from all tapasin complexes as shown in Fig. 7 ▸(*a*). The loop hovers above the α1 and α2–1 helices of MHC-I except for 6eny. The conformation of the loop varies from structure to structure, which indicates its mobility and flexibility. The α2–1 helix is drawn towards tapasin resulting in a widened peptide groove and reflecting the function of the chaperone. We also observed that the loops Gln189–His195 of tapasin and the loops Gln209–Gln215 of TAPBPR reveal varying conformations. Fig.7 ▸(*b*) shows the loops Gln189–His195 of tapasin extending underneath the peptide groove and interacting with β7 and β8 of MHC-I. Tapasin structures determined without MHC-I (PDB entries 7tuf and 3f8u) reveal this loop occupies a higher position. When tapasin in complexed with MHC-I (*e.g.* PDB entries 7qng, 7qpd, 7tue and 6eny), the loop is pushed down by about 5–10 Å, indicating its flexibility and plasticity. This loop appears to be vital in stabilizing the empty peptide groove (Natarajan *et al.*, 2018 ▸, 2019 ▸). Note that tapasin and TAPBPR, as shown in Fig. 7 ▸(*c*), differ in the length of this loop, as tapasin [Pro69–Ser110 (40 aa)] is much longer than that of TAPBPR [Cys101–Gln126 (26 aa)]. Additionally, tapasin has a unique interaction between Ser82 and the loop connecting β7 and β8 of MHC-I (Ser131).

**Figure 8:**
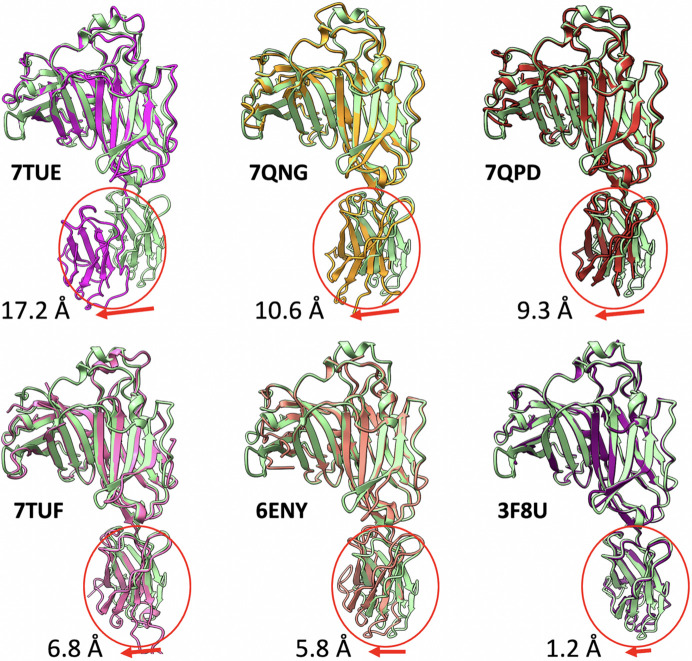
Domain movements of the chaperone tapasin. Tapasin from each complex is superimposed on the top domains (N and IgV domain, residues 2–280) of the *AlphaFold2* predicted model (green) as a reference point. The measured distance is between the L293 Cα atoms in the IgC domain, as shown by the number in the figure. The *AlphaFold2* model is similar to PDB entry 3f8u.

Dynamic domain movements accompany the interactions between the chaperones and MHC-I. Comparison of the tapasin/MHC-I complex (PDB entry 7tue) with the unchaperoned MHC-I (PDB entry 7tud) reveals that the α3 and β_2_m domains of the MHC-I and the IgC domains of the chaperone undergo positional reorientations. For example, as shown in Fig. 6 ▸(*a*), the IgC domain of tapasin swings up as much as 16 Å (Jiang *et al.*, 2022 ▸). These α3, β_2_m and IgC domain movements likely contribute to forming a stabilized peptide receptive binding groove. We compared the domain orientations in different complexes bound to tapasin: PDB entries 7qng (tapasin/D^b^/ERp57), 7qpd and 6eny (tapasin/A3 in PLC), 7tuf (tapasin with antibody Fab fragments PaSta1), and 3f8u in Fig. 8 ▸. The IgC domains of the different structures reveal movements varying from 1.2 to 17.2 Å (Fig. 8 ▸). Interestingly, tapasin that does not complex with MHC-I (*e.g.* PDB entries 7tuf and 3f8u) showed less movement. In contrast, movement seems more extensive when the tapasin is in complex with MHC-I (PDB entries 7tue, 7qng and 7qpd). Thus, the domains of tapasin may move differently by changing the hinge angle. We also consistently observed the domain movements in the complex of TAPBPR/H2-D^d^ (Jiang *et al.*, 2017 ▸).

### 5.2. ‘Negative allostery’ and ‘peptide trap’ mechanisms revealed by NMR

The dynamics of the chaperone/MHC-I peptide-exchange process were further investigated using solution NMR (McShan *et al.*, 2018 ▸). Using isotope-labeled MHC-I (H2-D^d^) and β_2_m, the authors observed conformational changes that were stabilized by TAPBPR interactions on decreasing peptide occupancy. The results demonstrate an inverse relationship between MHC-I peptide occupancy and TAPBPR binding affinity and support a ‘negative allostery’ model wherein structural features of transiently bound peptides control the regulation of a conformational switch near the TAPBPR binding site, triggering TAPBPR release. In complementary additional studies, McShan *et al.* (2021 ▸) studied the Gly24–Arg36 loop of TAPBPR and the analogous Glu11–Lys20 loop of tapasin using a combination of deep mutagenesis, isothermal titration calorimetry (ITC), fluorescence polarization (FP)-based assays and NMR methods. The results suggested that this loop of TAPBPR and tapasin contributes to a ‘peptide trap’ hovering above the MHC-I groove to restrain the dissociation of weak-binding peptides. This model does not suggest a short helix nor support direct interaction of this loop either with the F-pocket of the groove or directly with bound peptide.

### 5.3. The mechanism of peptide exchange in antigen presentation

There are three important interaction regions in chaperone/MHC-I complexes [Fig. 5 ▸(*c*)]. (1) The loop that links β1 and β2 strands (Glu11–Lys20 for tapasin, Ala25–Glu34 for TAPBPR) and hovers over the α1 and α2–1 helices of MHC-I. In the tapasin complex, Glu11 or Asp12 of β1 interacts with Ile142 of α2–1, and Lys20 of strand β2 interacts with Asn86 of α1 that holds the N domain of tapasin at the C-terminus of the peptide groove of MHC-I. (2) The strand β4 and the following loop Glu72 to Lys84 of tapasin interact with α2–1 and β8 where Glu72 and Ser74 of tapasin formed a pair of hydrogen bonds with Arg145 of the MHC-I α2–1 helix. Ser82 of tapasin interacts with Arg151 of α2–1 and Ser131 (between β7 and β8). Similarly, this interaction was also observed in the tapasin/ERp57/H2-D^b^ structure (PDB entry 7qng). Compared with tapasin, TAPBPR has a shorter loop after β4, and the α2–1 helix primarily interacts with β4. (3) The loop of Gln189-His195 of tapasin [Fig. 6 ▸(*b*)] or Gln209-Gln215 of TAPBPR extends underneath the peptide groove to interact with the β6 and β7 of MHC-I to stabilize the empty F-pocket. As shown in Fig. 5 ▸(*c*), several hydrogen bonds are observed between G192–H195 of tapasin and Arg111, Tyr113, Asp122 and Asn127 of HLA-B*44:05. In addition to these three regions, the domain movements [Figs. 6 ▸(*a*) and 8 ▸] of the membrane-proximal IgC domain, β_2_m and α3 of MHC-I result in a tightly packed trimer that undergirds the platform domain. Al­together, the three interaction regions and the domain movements provide the dynamic conformational changes on MHC-I and stabilize the peptide receptive state. Earlier studies (Fisette *et al.*, 2016 ▸) using molecular dynamics (MD) simulations already showed that the widening of the peptide groove on tapasin binding (particularly α2–1 and β7, β8) resulted from multiple interactions rather than being dominated by any single interaction alone. (Fisette *et al.*, 2016 ▸).

**Figure 9:**
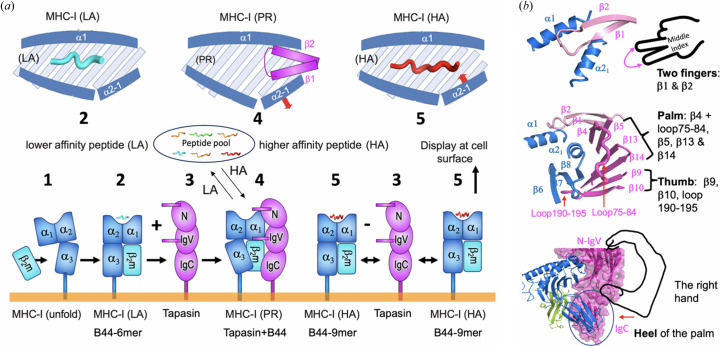
Structural mechanism of peptide exchange and illustration. (*a*) Structural mechanism of peptide exchange indicating dynamic intermediate states. State 1 indicates β_2_m binding to the heavy chain of MHC-I. State 2 shows the early loading of low-affinity peptides. State 3 displays the initial interaction of tapasin binding to MHC-I. State 4 summarizes the release of the lower-affinity peptide and formation of a peptide receptive complex. State 5 illustrates the binding of a high-affinity peptide, releasing MHC-I from the PLC. (*b*) The Finger–Palm–Heel model: a mechanical principle [This figure is modified from one previously published by Jiang *et al.* (2022 ▸) distributed under a Creative Commons CC BY 4.0 licence (https://creativecommons.org/licenses/by/4.0/)].

Fig. 9 ▸(*a*) illustrates a model for the progression of different steps during MHC-I peptide exchange. Initially, β_2_m binds to the heavy chain of MHC-I (state 1) that assists folding as a heterodimer of the complex, which may load some low-affinity peptides (state 2). The example of the structure of HLA-B*44:05-6-mer (PDB entry 7tud) may represent this state of MHC-I. On binding the chaperone tapasin (state 3), the MHC-I releases the lower-affinity peptide and forms a peptide-receptive tapasin/MHC-I complex (state 4). The structure of tapasin/HLA-B*44:05 (PDB entry 7tue), tapasin/H2-D^b^/ERp57 (PDB entry 7qng) and PLC (PDB entries 6eny and 7qpd) represent this state. When an optimal (high-affinity) peptide loads onto the peptide receptive state of MHC-I, the α2–1 helix is pulled back from the chaperone, and MHC-I enters a stable state (state 5) bound to a high-affinity peptide, represented by HLA-B*44:05-9-mer (PDB entry 7tuc; Jiang *et al.*, 2022 ▸). MHC-I with an optimal or high-affinity peptide can then traffic effectively through the Golgi, reach the cell surface, and then be displayed there for recognition by TCR of CD8^+^ cells or NK cell receptors.

The structural characterizations described above explain the low-affinity release mechanism and binding of higher-affinity peptides as catalyzed by chaperone function. We may interpret this mechanism as a human hand-grasp principle based on the ‘peptide trap’ model (McShan *et al.*, 2021 ▸). As shown in Fig. 9 ▸(*b*), β1 and β2 of the chaperone represent two fingers (index and middle); the palm consists of β4, loop 75–84, β5, β13 and β14. Additionally, β9 and β10 can be described as the thumb, and the heel of the palm is the IgC domain. The two fingers allow peptide exchange in the transition from the ‘closed’ to the ‘open’ form. The mechanism described above is similar for tapasin/MHC-I and TAPBPR/MHC-I. However, minor differences may exist (*i.e.* the precise interaction sites and loop functions). Precise details of TAPBPR or tapasin interactions may differ for different MHC-I alleles and clearly influence the MHC-I peptidome. Recent studies in mass spectrometric determination of HLA bound peptides have not only identified the motifs that are preferred by many MHC-I allelomorphs (Sarkizova *et al.*, 2020 ▸), but have extended our understanding of the role of tapasin in modulating the HLA-B*44:05 peptide repertoire (Kaur *et al.*, 2023 ▸).

## 6. Discussion of the TAPBPR Ala28–Glu37 and tapasin Glu11–Lys20 loops

There has been an ongoing discussion concerning whether a ‘scoop loop’ encompassing Ala28–Glu37 of TAPBPR (Sagert *et al.*, 2020 ▸; Thomas & Tampé, 2017 ▸) or individual residues Leu30 of TAPBPR (Ilca *et al.*, 2018 ▸) or Leu18 of tapasin (Lan *et al.*, 2021 ▸) actively compete for the binding of low-affinity peptides.

The cryoEM map and the model (PDB entry 7qpd) for the PLC have been improved (Domnick *et al.*, 2022 ▸) and the authors now designated this region (the loop of Glu11–Lys20 in tapasin) as an ‘editing loop’. The current model shows that this loop hovers over the α1 and α2–1 helices of MHC-I, consistent with PDB entries 7qng (tapasin/ERp57/MHC-I) and 7tue (tapasin/HLA-B44). As shown in Fig. 6 ▸(*a*), the loop Glu11–Lys20 from various complexes looks similar but flexible. Leu18 has been proposed as a critical residue in the ‘editing loop’ (Domnick *et al.*, 2022 ▸; Lan *et al.*, 2023 ▸, 2021 ▸; Müller *et al.*, 2022 ▸). In the tapasin/HLA-B*44:05 structure (PDB entry 7tue), the MHC-I residues Thr80 and Tyr84 of α1 together with Lys146 and Ile142 of α2–1 form a ‘lock’ that prevents the tapasin Glu11–Lys20 loop from interacting with the F-pocket (Jiang *et al.*, 2022 ▸). In MD simulations of tapasin (Fisette *et al.*, 2020 ▸, 2016 ▸), the loop Glu11–Lys20 swings away and does not approach the F-pocket, although this loop shows high fluctuation.

## 7. Conclusions

This topical review summarizes the structural immunology of MHC-I molecules with a focus on the role of chaperones in antigen presentation. We also review the strategies for obtaining the chaperone/MHC-I complexes *in vitro*. Specifically, we have described recently obtained structures (X-ray, cryoEM) of chaperone/MHC-I complexes. We provide an overview of the structural mechanism of peptide loading and exchange in MHC-I. Most importantly, we now recognize major regions of MHC-I/chaperone interaction and identify domain movements that govern conformational changes of the peptide groove that control the release of low-affinity peptide and stabilize the peptide receptive state for exchange with high-affinity peptide. In general, tapasin/MHC-I and TAPBPR/MHC-I share the same structural mechanism of peptide loading and exchange, although there are minor differences in the details of the specific loops and residues used by the two chaperones.

Our current understanding of the structural basis of chaperone function in antigen presentation derives from a host of studies based on cell genetics, biochemistry, immunology and structural biology. The molecular details that we now appreciate not only serve to satisfy our scientific curiosity, but also form a factual basis to aid in the design of MHC-peptide complexes that may be used for experimental, diagnostic and therapeutic applications.

## Figures and Tables

**Table 1:** Summary of structures of MHC-I chaperones and their complexes

Complex	PDB entry	Resolution (Å)	X-ray/cryoEM	Reference
Tapasin/ERp57	3f8u	2.6	–	(Dong *et al.*, 2009 ▸)
TAPBPR/H2-D^d^	5wer	3.4	–	(Jiang *et al.*, 2017 ▸)
TAPBPR/H2-D^b^	5opi	3.3	–	(Thomas & Tampé, 2017 ▸)
Tapasin/HLA-B44*05	7tue	3.1	–	(Jiang *et al.*, 2022 ▸)
Tapasin/PaSta1	7tuf	2.8	–	(Jiang *et al.*, 2022 ▸)
Tapasin/PaSta2	7tug	3.9	–	(Jiang *et al.*, 2022 ▸)
Tapasin/ERp57/H2-D^b^	7qng	2.7	–	(Müller *et al.*, 2022 ▸)
PLC (tapasin/HLA-A*03)	6eny	5.8	EMD-3906	(Blees *et al.*, 2017 ▸)
PLC (tapasin/HLA-A*03)	7qpd	3.7	EMD-14119	(Domnick *et al.*, 2022 ▸)
